# Clinical and molecular findings in two siblings with neuroblastoma-amplified sequence (NBAS) deficiency corroborates a more severe phenotype for compound-heterozygous truncating and splice variants in *NBAS*, in response to Silverstein et al. [1]

**DOI:** 10.1016/j.ymgmr.2026.101366

**Published:** 2026-09-30

**Authors:** Michael Schlotterbek, Ute Grasshoff, Sarah Baumann, Christoph Kernstock, Ludger Schöls, Tobias B. Haack, Lena Zeltner

**Affiliations:** a Institute of Medical Genetics and Applied Genomics, University of Tuebingen, Tuebingen, Germany; b Department of Neurodegenerative Diseases, Hertie-Institute for Clinical Brain Research and Center of Neurology, University of Tuebingen, Tuebingen, Germany; c University Eye Hospital, Centre for Ophthalmology, University of Tuebingen, Tuebingen, Germany

Dear Editor,

We read with great interest the article *“RNA sequencing driven diagnosis expands the phenotypic spectrum of NBAS deficiency”* by Silverstein et al. in *Molecular Genetics and Metabolism*, expanding the multisystemic phenotype associated with biallelic *NBAS* variants [1]. Their patient carrying a compound-heterozygous truncating and splice variant, both predicted to result in nonsense-mediated mRNA decay (NMD), provides further insight into the complex genotype-phenotype-correlation in *NBAS*-deficiency, especially in light of previously lacking reported patients with biallelic loss-of-function variants [2].

We would like to contribute our clinical and molecular findings in two siblings carrying the same splice variant previously reported by Silverstein et al., and a compound-heterozygous frameshift variant in *NBAS.* Both presented with primordial dwarfism, syndromic progeroid facial features and developmental delay (Fig. 1b, c). At time of clinical evaluation, the 48-year-old brother and 43-year-old sister showed severe short stature (height: 100 cm and 115 cm, respectively) and microcephaly (head circumference: 46 cm and 45 cm). The sister was more severely affected, with severe global developmental delay, speech limited to short sentences, whereas the brother was able to communicate complex content grammatically correct. Both showed severe spastic tetraparesis with contractures, being wheelchair-dependent. Additional shared findings included reduced visual acuity, retinal dystrophy, reduced elasticity of skin, osteoporosis and high-pitched voice. Peripheral blood smear analysis showed Pelger-Huët-anomaly. The brother also had a congenital bladder exstrophy.

**Fig. 1:**
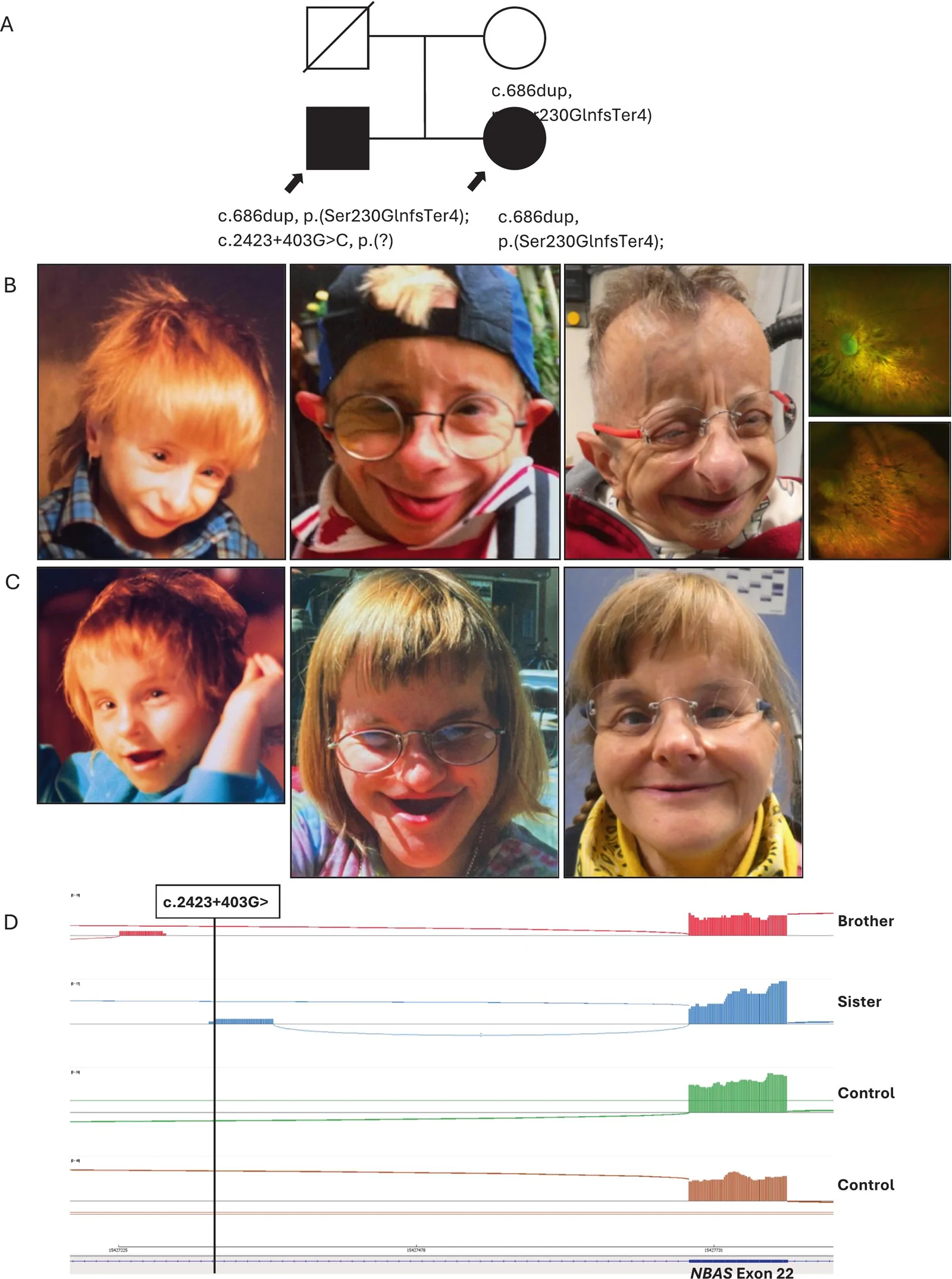
(A) Pedigree and reported *NBAS* variants. (B) Photographs of the affected brother in childhood, at age 14 years, at evaluation (age 48 years), fundus photographs of the left (upper) and right (lower panel) eye showing retinal dystrophy. (C) Photographs of the affected sister in childhood, at age 10, and at evaluation (age 43). Note syndromic facies with progeroid appearance, pointed nose, prominent triangular chin, wide mouth with thin vermilion borders, low-set and protruding, dysmorphic ears and deep-set eyes. (D) RNAseq from patient fibroblast, hinting at a splice effect with inclusion of intronic sequence of Intron 22 in *NBAS*.

Multi-sample genome sequencing revealed biallelic *NBAS* (NM_015909.4) variants in both siblings, a maternally inherited frameshift variant (c.686dup, p.(Ser230GlnfsTer4)) and the splice variant (c.2423 + 403G > C, p.(?)). Long-read genome sequencing established biallelic phasing of the variants in both affected siblings. RNA-sequencing from patient fibroblasts indicated significantly reduced (z-score − 3334 and − 3378 respectively, *p*-value <0.001) *NBAS* mRNA levels compared to in-house controls, in line with partial NMD. Visualization in IGV hinted at a splice event with reads showing inclusion of intronic sequence, in line with the reported pseudoexonic inclusion in intron 22 (Fig. 1d). Used sequencing methods have been described previously [3], [4].

Our cases support the previously reported genotype-phenotype-association of a more severe phenotype associated with a truncating variant *in trans* with a recurrent splice variant in *NBAS*, further corroborating the broader phenotypic spectrum of *NBAS* deficiency.

## CRediT authorship contribution statement

**Michael Schlotterbek:** Conceptualization, Investigation, Writing – original draft, Data curation. **Ute Grasshoff:** Investigation, Writing – review & editing, Supervision. **Sarah Baumann:** Investigation, Data curation. **Christoph Kernstock:** Investigation. **Ludger Schöls:** Writing – review & editing, Supervision. **Tobias B. Haack:** Supervision, Writing – review & editing, Data curation. **Lena Zeltner:** Investigation, Writing – review & editing.

## Ethics declaration

Written informed consent to take part in the study and to publish the article has been obtained from all participants or their legal representatives. The privacy rights of participants have been observed.

Informed consent for scientific publication including pictures of affected family members was provided by the legal guardian.

This study included organ or tissue donors. This study includes human biological material and consent was obtained by donors, or their next of kin or legal representatives, for use in this study and for publication of the article. The samples used in this research were not sourced from executed prisoners or prisoners of conscience.

This study was performed in compliance with relevant laws, regulatory frameworks and guidelines where the research took place. This study was approved by the Ethics Committe Tübingen. (Approval No. 432/2023BO1).

This research follows the CARE guidelines and the CARE checklist.

## Funding

T.B.H. received funding from the European Commission (Recon4IMD - GAP-101080997) and the German Research Foundation (DFG / EJP-RD Artemis: 542553983). M.S., L.S. and T.B.H. are supported by the Clinician Scientist program “PRECISE.net 2.0” funded by the 10.13039/501100003042Else Kröner-Fresenius-Stiftung.

## Declaration of competing interest

No author has any competing interests.

## Data Availability

Data will be made available on request.

## References

[1] Silverstein S., Cassini T., Fu J., Pusey B., Macnamara E., Frost F.G. (2025). RNA sequencing driven diagnosis expands the phenotypic spectrum of NBAS deficiency. Mol. Genet. Metab..

[2] Staufner C., Peters B., Wagner M., Alameer S., Barić I., Broué P. (2020). Defining clinical subgroups and genotype-phenotype correlations in NBAS-associated disease across 110 patients. Genet. Med..

[3] Menden B., Incebacak Eltemur R.D., Demidov G., Sturm M., Park J., Huridou C. (2026). Loss-of-function variants in the CAPN1 activator CD99L2 cause X-linked spastic ataxia. Nat. Commun..

[4] Figueroa K.P., Gross C., Buena-Atienza E., Paul S., Gandelman M., Kakar N. (2024). A GGC-repeat expansion in ZFHX3 encoding polyglycine causes spinocerebellar ataxia type 4 and impairs autophagy. Nat. Genet..

